# Efficacy of Ionization-Adjusted PN Filler in Minimizing Injection Pain: A Randomized, Patient-Blinded, Split-Face Study

**DOI:** 10.1007/s00266-025-05320-x

**Published:** 2025-12-08

**Authors:** Woosang Jeon, Minwoo Park, Sug Won Kim, Jiye Kim

**Affiliations:** Department of Plastic and Reconstructive Surgery, Wonju Severance Christian Hospital, Wonju College of Medicine, Yonsei University, 20 Ilsan-ro, Wonju-si, Gangwon-do, Wonju, Republic of Korea

**Keywords:** Polynucleotide filler, Ionization adjustment, Injection pain, Skin booster, Aesthetic dermatology, Split-face trial

## Abstract

**Abstract:**

Polynucleotide (PN) fillers are widely used for skin rejuvenation due to their regenerative properties; however, injection-related pain remains a major drawback. This randomized, patient-blinded, split-face study evaluated a novel “ionization-adjusted” PN filler with modified pH and ionic strength designed to reduce injection pain without compromising efficacy. Fifteen participants received 1 mL of conventional PN filler on one side of the face and 1 mL of the ionized PN filler on the other. Pain was assessed using a 10-point Visual Analog Scale (VAS), while wrinkle improvement was evaluated using the Crow’s Feet Grading Scale (CFGS) and Global Aesthetic Improvement Scale (GAIS). The ionized PN filler demonstrated significantly lower pain scores (VAS 3.39 ± 1.81) compared to the conventional filler (VAS 5.04 ± 2.19, p < 0.005), with fewer cases of erythema (40% vs. 60%) and embossing (13.3% vs. 56.7%). Both formulations showed significant improvements in CFGS and GAIS scores without statistical differences. These findings suggest that ionization-adjusted PN filler provides a more comfortable injection experience while maintaining similar clinical efficacy and safety profiles.

**Level of Evidence I:**

This journal requires that authors assign a level of evidence to each article. For a full description of these Evidence-Based Medicine ratings, please refer to the Table of Contents or the online Instructions to Authors www.springer.com/00266.

## Introduction

The development of wrinkles around the eyes, forehead, cheeks, and lips represents a natural progression of aging, yet these wrinkles often contribute to a fatigued and aged appearance. The occurrence of wrinkles is result to skin aging. Wrinkle formation is closely associated with skin aging. Aged skin loses its elasticity and becomes thinner due to the depletion of dermal collagen. Additionally, it loses its ability to recover from changes in facial expressions and the effects of gravity, leading to the easy formation of wrinkles [1, 2].

The fillers containing polynucleotide (PN) have emerged as popular injectable treatments for skin rejuvenation and anti-aging. PN is typically derived from purified DNA fragments (polydeoxyribonucleotide) of trout or salmon, and when injected into the dermis, it can stimulate fibroblast activity, promote collagen synthesis, and aid tissue regeneration [3–5]. Clinical studies have shown improvements in skin texture, elasticity, and hydration following PN filler treatments [6]. This makes PN fillers effective as “skin boosters” to improve fine wrinkles, skin thickness, and overall skin appearance [7–10].

Despite their regenerative benefits, a major limitation of PN fillers is the significant pain associated with the injection procedure [11]. PN treatments involve a series of multiple intradermal injections (often delivered as micro-droplets across the face), which can be highly uncomfortable even with prior topical anesthesia. The pain can cause patients to move or tense up during the procedure, potentially leading to imprecise filler placement or bruising. Indeed, patient tolerance is a critical factor; if the injections are too traumatic, patients may be unwilling to continue or repeat treatments. Thus, minimizing injection pain is crucial to ensure patients can complete the recommended treatment regimen and achieve optimal results.

Various strategies have been explored to mitigate injection pain in cosmetic treatments. For hyaluronic acid (HA) dermal fillers, the inclusion of lidocaine (0.3%) in the filler has become a standard practice since the mid-2000s to improve patient comfort [12]. This addition significantly decreases pain during and after filler injections and enhances patient satisfaction. However, PN fillers on the market generally do not contain lidocaine, and physicians typically rely on topical anesthetic creams or nerve blocks to manage pain. Another approach to reduce pain is to change the delivery method. A recent study by Park et al. compared conventional needle injections of PN filler with a needle-free jet injector system in a split-face trial [11]. The needle-free device delivered PN into the dermis using high-pressure jet streams, resulting in significantly lower pain (VAS 2.9 vs. 5.4) and higher patient satisfaction, as well as equivalent or superior skin improvements, compared to manual needle injection. While needle-free injection is promising, such devices may not be readily available in all clinics, and some practitioners continue to prefer the control of manual injections. Therefore, optimizing the filler itself to reduce pain would be highly valuable.

The causes of injection pain with PN fillers are multifactorial, but the key factors regulating injection pain are viscosity, pH, and osmolarity of formulations. It is well known that the solutions showing lower pH and lower osmolarity than physiologic condition (pH 7.4 and 280~290 mOsmol/kg) can exacerbate pain at the injection site [13, 14]. In analogy, buffering local anesthetic solutions to a neutral pH significantly reduces injection pain by reducing acidity.

It is very interesting that even though the pH and osmotic pressure of commercially available PN fillers are designed to meet physiologic condition (pH 7.2–7.5, 280–320 mOsmol/kg), the pain observed when commercially available PN fillers are injected into skin tissue is considerably high. Thus, we thought that the injection pain of PN fillers is not suppressed by commonly accepted formulation design near physiologic condition, and hypothesized that the injection pain of PN fillers is derived from microscopic osmotic pressure change during PN filler injection. This hypothesis is based on the dissociation tendency of sodium (Na) ionically bound to orthophosphate in PN. Basically, the pKa of sodium bound to orthophosphate (PO_4_^−3^) is about 12. Thus, Na dissociation in PN is not complete in physiologic pH, and the Na dissociation is additionally suppressed by high concentration in PN filler as much as 2 w/v%. When PN filler is introduced into skin tissue, the concentration of PN rapidly decreased, and the dissociation of Na is enhanced, and this Na dissociation in PN can reveal microscopic osmolality increase. Thus, we designed novel PN formulation refer to “an ionization adjusted PN filler” its pH is tuned to 7.6–7.8 to evade hazard relating high pH over 8.0 but to enlarging Na dissociation ratio. The osmolality of the ionization-adjusted PN filler was designed to meet physiologic osmolality.

The present study is a randomized, patient-blinded, split-face trial designed to investigate the efficacy of the ionization-adjusted PN filler in reducing injection pain, compared to the conventional PN filler. Additionally, the study evaluates whether the new formulation maintains comparable clinical outcomes in terms of skin rejuvenation. We measured immediate pain responses as well as short-term (4 week) outcomes on each side of the face. Our hypothesis was that the ionization-adjusted PN filler would significantly reduce pain during injection while achieving skin improvement outcomes non-inferior to the standard PN filler. Through this study, we aim to provide evidence for a simple yet impactful modification to improve the tolerability of PN filler treatments.

## Materials and Methods

### Study Design and Subjects

This study was designed as a single-center, randomized, patient-blinded, split-face trial. To conduct this study, a total of 4 injections were administered according to the PN filler usage protocol for periorbital wrinkle improvement in 15 patients, resulting in the collection of pain data from a total of 60 injection sessions. A total of 15 patients seeking improvement in periorbital wrinkles participated in this study, and patients with a pre-procedure Crow’s Feet Grading Scale (CFGS) score of 2–4 were selected.

This study was conducted in accordance with the ethical guidelines outlined in the Korean Good Clinical Practice (KGCP) and the Declaration of Helsinki. The protocol and all associated documents, including the initial and any revised informed consent forms, were reviewed and approved by the Institutional Review Board (IRB) prior to implementation (CR223013). Written informed consent was obtained from all participants, ensuring their understanding of the study’s purpose, procedures, risks, and benefits. This consent process was documented thoroughly to comply with regulatory standards and to protect the rights and well-being of the participants.

### Study Device

Ionization PN filler HP Vitaran I (BR PHARM Co., Ltd., Wonju, Korea) was used as test group. Conventional PN filler Rejuran i (PharmaResearch Products, Inc., Seoul, Korea) was used as control group. 1 mL of PN filler and 1 mL of ionized PN filler were injected in each side of periorbital area.

### Treatment Protocol

At the baseline visit, screening and randomization of the subjects were performed. Participants were randomly assigned to receive treatment on their crow’s feet using up to 1.0 mL of the investigational device, HP Vitaran I, on one side and the control device, Rejuran i, on the other side. To minimize variability in application technique, procedures were performed by the same investigator. Adverse events were monitored for 30 minutes immediately following the application of the medical devices. After the initial application (week 0), three repeat treatments were administered at two-week intervals, resulting in a total of four treatments. During repeat treatments, the investigator applied the devices to the same area as the initial application, ensuring that the injection volume did not exceed the initial treatment’s 1.0 mL.

In this study, all patients received injections with a 34G needle and were blinded to the treatments by wearing eye masks to prevent them from seeing the devices. Because the filler’s pH was thought to affect pain perception, pain was assessed using the Visual Analog Scale (VAS), which served as the primary endpoint for comparing the devices.

**Fig. 1:**
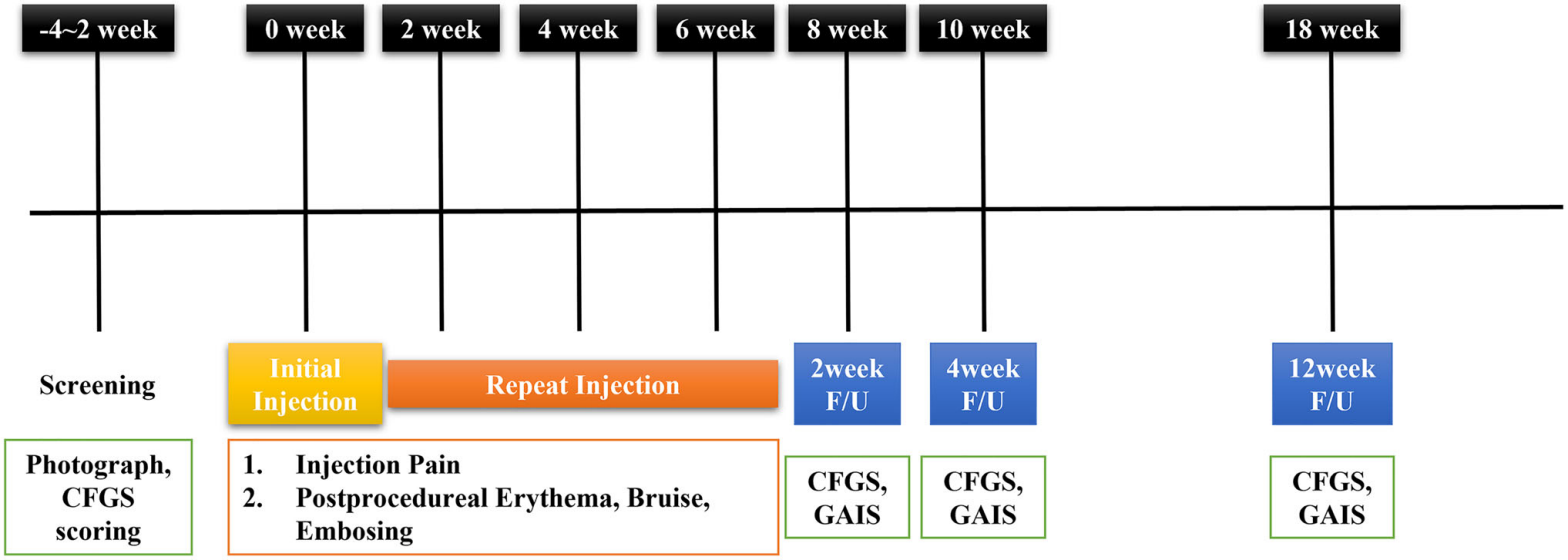
Flowchart of clinical study

Safety and efficacy assessments were conducted at 2 weeks, 4 weeks, and 12 weeks following the final application of the devices (Fig. 1).

### Pain Measures

Pain was assessed in 60 procedures immediately after treatment using the VAS. A 10 cm line was anchored with “no pain (0)” at the left end and “unbearable pain (10)” at the right end, and patients were instructed to mark their perceived pain level on the line. The distance from the left end to the patient’s mark was then measured in centimeters and recorded as the pain score.

### Efficacy Measures

Efficacy evaluation included the Crow’s Feet Grading Scale (CFGS) assessed by independent evaluators through photography that was obtained with Mark Vu skin diagnostic device (PSI plus, Suwon, Korea). Global Aesthetic Improvement Scale (GAIS) was evaluated by participants (1 = very much improved, 2 = much improved, 3 = improved, 4 = no change, 5 = worsened). Follow-up assessments were carried out for a duration of 12 weeks following the final injection.

### Safety Measures

All adverse events including pain, induration, swelling, redness, pruritus, and hemorrhage were evaluated at each visit. Additionally, all adverse events were documented for each subject, including an assessment of causality and severity. We also included measurements of laboratory tests, pregnancy tests, vital signs, and physical examinations. Any abnormal values were documented in relation to the clinical trial device, and follow-up investigations were conducted to assess causality.

### Statistical Analysis

Demographic data and safety evaluation metrics were analyzed using SAS® software (Version 9.4, SAS Institute, Cary, NC, USA). Comparative analyses between groups, as well as within-group assessments, were performed using two-tailed statistical tests with a significance level of *α* = 0.05. Effect sizes (Cohen’s d) were calculated based on the mean and standard deviation of the data. An a priori power analysis was conducted using PASS 2025 (v25.0.1; NCSS, LLC, Kaysville, UT, USA).

The primary objective of the power analysis was to confirm that the study had sufficient statistical power to detect a mean paired difference of 1.65 units, assuming a standard deviation of 2.42 and a two-sided paired t test. A sample size of 60 was determined to yield approximately 99.94% power (*α* = 0.05, *β* = 0.00061), corresponding to an effect size (Cohen’s d) of 0.68182. This level of power ensured a very high probability of detecting a true mean difference from zero.

## Results

### Patient Baseline Characteristics

Table 1 presents the demographic characteristics of the 15 subjects enrolled in the clinical trial. Twelve females and three males were included in this clinical trial. The mean age of the subjects was 43.9±6.5 years. Without drop out, all the subject completed this clinical trial. None of the participants had a history of prior filler or botulinum toxin treatments, nor did any show allergic reactions to PDRN or PN. Past medical history included Hypertension (*n*=3) and Hypercholesterolemia (*n*=1).

**Table 1:** Baseline demographics of patient

Variable	Value
Age, mean±SD (years)	43.9±6.5
Sex (n, %)	
Female	12(80)
Male	3(20)
BMI	24.66
Prior filler injection	0
Prior botulinum toxin injection	0
Baseline Crow's Feet Grade, n (%)	
Grade 4	1
Grade 3	5
Grade 2	9
Allergic reaction to PDRN/PN	0
Past History	
Hypertension (HTN)	3
Hypercholesterolemia	1
Diabetes mellitus (DM)	0

### Pain Score Evaluation

**Fig. 2:**
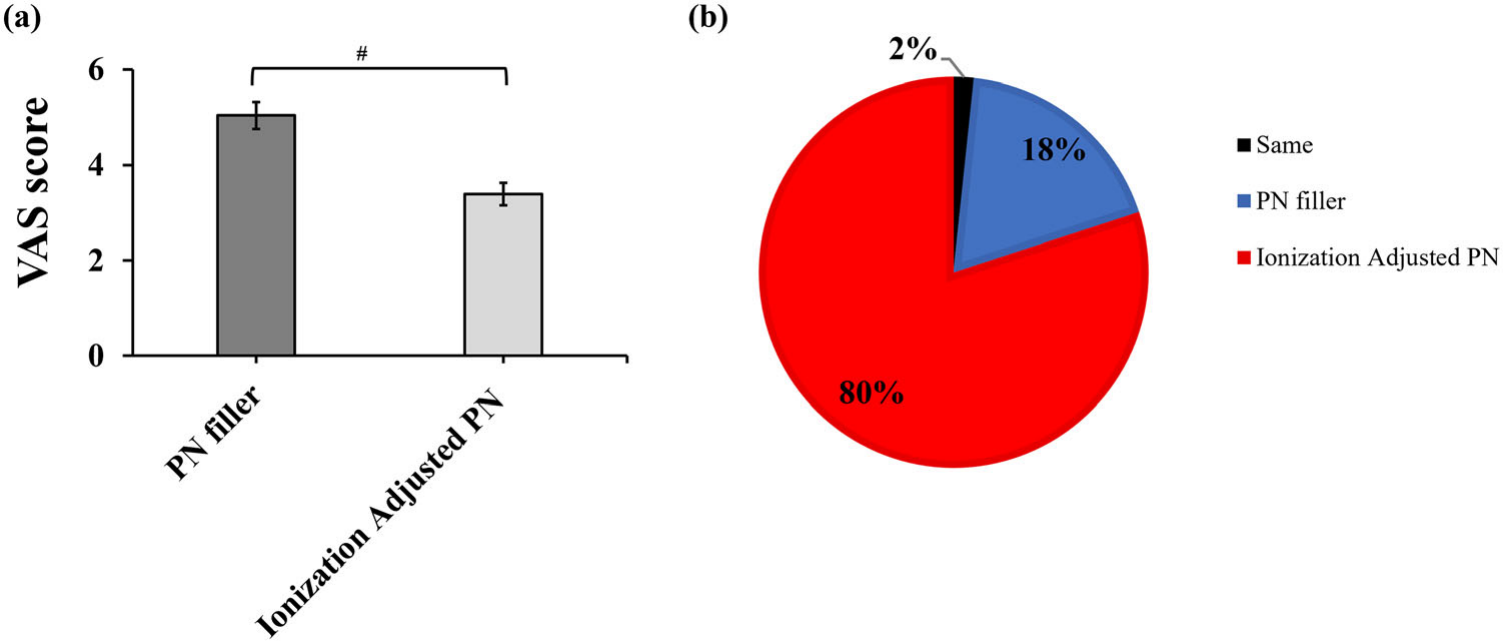
**a** Comparison of mean VAS (Visual Analog Scale) injection pain scores between conventional PN filler and ionization-adjusted PN filler. An a priori power analysis was conducted using PASS 2025 (v25.0.1; NCSS, LLC, Kaysville, Utah, USA), yielding 99.9% power for the VAS scores. **b** Distribution of comparative pain perception across 60 procedures. #*p* < 0.0001, Wilcoxon signed-rank test

All participants were able to provide independent pain ratings for each side of the face. A clear and statistically significant difference in injection pain was observed between the two filler formulations. The mean VAS pain score for the conventional PN filler was 5.01 ± 2.19, whereas the ionization-adjusted PN filler demonstrated a lower mean score of 3.39 ± 1.81. The difference was statistically significant (*p* < 0.0001, Fig. 2a), with a large effect size (Cohen’s *d* = –0.92), indicating a substantial reduction in adverse outcomes. The 95% confidence intervals (CIs) for the means were 4.48–5.61 and 3.20–3.74, respectively.

**Fig. 3:**
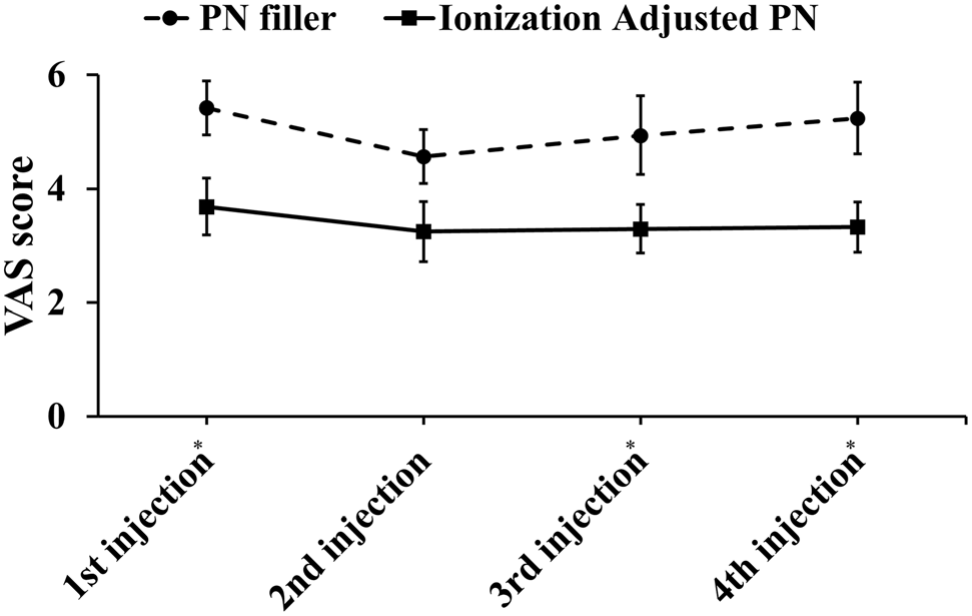
Injection pain score at each treatment period, *p < 0.05, paired t test

In a total of 60 procedures, the test formulation resulted in lower pain scores in 48 cases (80%), whereas the control was superior in 11 cases (18.3%) (Fig. 2b). Evaluation of pain scores across repeated treatment sessions revealed a slight decrease in pain during the second injection. Overall, pain scores varied significantly across subsequent treatments, except during the second session. Consistently, the ionization-adjusted PN filler produced lower pain scores at each injection compared to the conventional filler (Fig. 3), suggesting that the difference in pain perception was noticeable to the patients.

### Post-Procedural Erythema, Bruise and Embossing

In 60 procedures, post-procedural erythema was observed 35 cases(60%) of PN filler group and 24 cases (40%) was in ionization-adjusted PN filler group. Mean times for erythema disappear were 1.37±1.68 days in PN filler group and 1.13±1.79 days in ionization-PN filler group (Table 2).

**Table 2:** Observation of post-procedural erythema, bruise, embossing

		PN filler (60 cases)	Ionization-Adjusted PN filler (60 cases)	*p* value
Erythema	Frequency of erythema	36 (60%)	24 (40%)	0.0075^d^
	Times for erythema disappear (days) (mean ± SD)	1.37±1.68	1.13±1.79	0.1199^b^
Bruise	Frequency of bruise	22 (36.67%)	14 (23.33%)	0.0963^d^
	Times for bruising disappear (days) (mean ± SD)	1.93±3.41	1.33±3.10	0.0708^b^
Embossing	Frequency of embossing	34 (56.67%)	8 (13.33%)	<.0001^c^

^b^Wilcoxon signed-rank test, ^c^McNemar test, ^d^exact McNemar test

Post-procedural bruising was observed in 22 cases (36.67%) in the PN filler group, with a mean duration of 1.93 ± 3.41 days, and in 14 cases (23.33%) in the ionization-adjusted PN filler group, with a mean duration of 1.33 ± 3.10 days. Embossing occurred in 34 cases (56.67%) in the PN filler group, compared to 8 cases (13.33%) in the ionization-adjusted PN filler group. Post-procedural erythema, bruising, and embossing were more frequently observed in the PN filler group.

The frequency of erythema (*p* = 0.0075) and embossing (*p* < 0.0001) showed statistically significant differences between the two groups. However, both the frequency and duration of bruising did not differ significantly between the groups.

### Safety Evaluation

Among the 15 subjects (60 procedures), most adverse events (AEs) were mild, tolerable, and confined to the injection sites, manifesting as erythema, bruising, and embossing. No systemic side effects were observed in any of the cases. (Table 3)

**Table 3:** Summary of adverse events reported in 60 procedures

Adverse Event	PN filler side	Ionization-Adjusted PN filler side
Local adverse event		
Erythema	36	24
Bruise	22	14
Embossing	34	8
Systemic adverse event	0	0

### CFGS and GAIS

The average of baseline CFGS of PN filler group was 2.33 ± 0.82 and of ionization-adjusted PN filler group was 2.33 ± 0.82. Two weeks after injection, CFGS was significantly improved than baseline in both of PN filler and ionization-adjusted PN filler group. In the PN filler group and ionization-adjusted PN filler group, there was no statistically difference of CFGS. Improved CFGS was maintained through the 12 weeks after injection.

GAIS scores were self-assessed by study participants. The average GAIS score of ionization-adjusted PN filler was 1.67 ± 0.98 in 2 weeks follow-up, 1.73 ± 1.22 in 4 weeks, 1.6 ± 0.91 in 12 weeks, and average GAIS score of PN filler was 1.33 ± 0.82 in 2 weeks follow-up, 1.47 ± 0.99 in 4 week, 1.67 ± 0.98 in 12 weeks (Table 4).

**Table 4:** Mean changes in Crow’s Feet Grading Scale (CFGS) and Global Esthetic Improvement Scale (GAIS)

	CFGS				GAIS	
	PN filler	*p* value	Ionization-Adjusted@PN filler	*p* value	PN filler	Ionization-Adjusted PN filler
Baseline	2.33±0.82		2.33±0.82			
2 week F/U	1.53±0.64	<0.001	1.6±0.63	<0.001	1.33±0.82	1.67±0.98
4 week F/U	1.53±0.64	<0.001	1.53±0.64	<0.001	1.47±0.99	1.73±1.22
12 week F/U	1.67±0.90	<0.00	1.67±0.90	<0.00	1.67±0.98	1.6±0.91

Compared with baseline CFGS: Wilcoxon test

## Discussion

The global skin booster market is gradually expanding. Although the term “skin booster” has not previously had a defined scope, it broadly encompasses substances that, when injected or applied to penetrate the dermis, contribute to skin rejuvenation [10]. Unlike conventional fillers, skin boosters are injected superficially, helping to slow the skin aging process and rebuild collagen levels. Among these skin boosters, polynucleotide (PN) fillers have recently attracted attention as biocompatible, biostimulatory dermal fillers [15]. PN fillers play an important role in the Korean aesthetic market as skin boosters due to their versatility in treating various indications, including fine periocular wrinkles, scars, and facial erythema. However, injection-related pain can be a significant barrier preventing patients from undergoing PN filler procedures. This study aimed to objectively evaluate injection pain associated with PN fillers using the Visual Analog Scale (VAS). Furthermore, we aimed to demonstrate the superiority of an ionization-adjusted PN filler designed to reduce injection pain compared to conventional PN fillers, utilizing a randomized, split-face comparative study design.

In this randomized split-face study, we demonstrated that adjusting the ionic composition of a PN filler significantly alleviates injection pain while preserving its aesthetic benefits. The ionization-adjusted PN filler resulted in markedly lower pain compared to the conventional formulation, confirming our primary hypothesis. Equally important, skin rejuvenation outcomes—as measured by physician-assessed CFGS and GAIS—were comparable between the two sides, indicating that the modification did not compromise the filler’s effectiveness. These results have practical implications for improving patient comfort during aesthetic treatments.

The rationale behind this observation lies in the chemical structure of the PN filler, wherein PO_4_^−3^ and Na^+^ are ionically bonded. In its pre-filled form within the syringe, this ionic bond remains largely undissociated. However, upon injection into biological tissue—where the filler interacts with plasma or lymphatic fluid—rapid dissociation of Na^+^ from PO_4_^−3^ is believed to occur. This sudden change in microscopic osmolality may trigger nociceptive responses, resulting in injection-associated pain [16].

To address this, we hypothesized that adjusting the pH of the PN solution within the syringe would promote the complete pre-injection dissociation of PO_4_^−3^ and Na^+^, thereby minimizing the osmolarity gap upon tissue administration [17–19]. Moreover, recent research in dermal injections has underscored the importance of formulation parameters, beyond needle size and injection technique, in modulating pain. Usach et al. [13] reported that isotonic solutions (≈300 mmOsm/kg) with physiological pH (~7.4) and low buffer strength (phosphate ≤10 mM) minimize nociceptor activation. In aesthetic practice, Alghonaim et al. [20] and Shin et al. [21] both showed that simple alkalinization of botulinum toxin reconstitution media (using sodium bicarbonate) yields clinically significant reductions in VAS scores compared to unbuffered saline. Our findings support this mechanism, as the modified filler formulation was associated with significantly reduced injection pain without compromising therapeutic efficacy.

Inspired by these findings, our ionization-adjusted PN filler is precisely formulated to physiological pH and near-isotonic osmolality within the syringe, ensuring that PO_4_^−3^ and Na^+^ are fully dissociated prior to administration. By pre-emptively closing the osmolarity gap at the time of injection, we achieved a marked decrease in pain scores without impairing collagen-stimulating efficacy.

Although post-injection erythema has not been definitively characterized, it is generally considered a transient reaction, presumed to be associated with residual animal-derived proteins present in the PN filler. Polydeoxyribonucleotide (PN) is a DNA polymer extracted from the testes of salmon or trout, and trace amounts of animal-derived proteins are inevitably present during the extraction process. These residual proteins may act as immunogenic substances once introduced into human tissue, potentially eliciting immune responses. Therefore, minimizing the content of such protein impurities is critical to reducing adverse reactions.

The current device significantly reduces protein impurity levels of that found in conventional PN fillers, which may account for the minimized erythema observed in this study. In addition, embossing, a phenomenon frequently observed by experienced practitioners of PN filler treatments, refers to transient, papular elevations of the skin (approximately 2–3 mm in size) at the injection site. This is attributed to the viscosity of the injected substance, which causes the filler to temporarily retain its shape within the dermal tissue. To mitigate this reaction, Vitaran I has been formulated with NaCl as an osmotic modulator, promoting rapid dispersion of the filler upon injection and thereby minimizing the likelihood and duration of such localized tissue reactions. Unlike the use of exogenous substances for osmotic control, the use of the biocompatible, physiologically derived NaCl is presumed to contribute to the reduction in embossing observed in the ionization-adjusted PN filler group.

Aside from minor local reactions such as post-injection embossing and erythema, no systemic adverse events were observed following PN filler administration. Upon administration, polynucleotide (PN) is enzymatically degraded by endogenous DNases, and the resulting products are either absorbed as nutrients or processed through the same physiological recycling pathways as endogenous nucleotides, nucleosides, nitrogenous bases, and phosphates before being reused or excreted. Therefore, systemic toxicity attributable to PN is unlikely [22, 23].

As this was a pilot clinical study, objective instrumental assessments such as cutometry or 3D imaging were not performed, which may limit the objectivity of the outcome evaluation. However, the assessment of wrinkle severity was conducted by an independent rater, distinct from the physician who performed the procedure, using the Crow’s Feet Grading Scale (CFGS). Furthermore, the universally recognized Global Aesthetic Improvement Scale (GAIS) was used to evaluate patient satisfaction.

A crucial aspect of this study was verifying that ionization adjustment did not interfere with efficacy; our results showed no compromise, as both formulations led to equivalent improvements in wrinkles at 2 weeks, confirmed by GAIS and objective measures indicating intact collagen-stimulating effects of PN. PN fillers also improved skin texture and reduced pore visibility. Safety evaluation indicated that both formulations had benign and transient post-injection effects typical of such procedures, without unexpected inflammation or immunogenic responses associated with the adjusted filler. However, the modest sample size (*n*=15), while sufficient to demonstrate significant differences in pain, might limit the detection of subtle efficacy differences or rare side effects, highlighting the need for larger trials to confirm these findings and thoroughly assess safety.

In conclusion, our findings demonstrate that simple modifications to the PN formulation can significantly enhance patient experience without compromising efficacy. This aligns with the broader trend in aesthetic medicine toward prioritizing patient comfort and safety. Minimizing pain is not merely about comfort; it directly influences treatment success and patient compliance. By adopting an ionization-adjusted PN filler, clinicians can achieve optimal skin rejuvenation outcomes with a markedly more tolerable procedure for their patients.

## Conclusions

In summary, this patient-blinded, split-face trial demonstrated that adjusting the ionic conditions of a PN filler significantly reduces injection pain without compromising its rejuvenating efficacy. The ionization-adjusted PN filler showed significantly lower pain scores compared to the conventional filler and achieved non-inferior outcomes in wrinkle improvement. These findings suggest that a physiologically optimized PN filler can enhance patient comfort and satisfaction, potentially improving compliance with multi-session treatments while delivering the same cosmetic benefits. Incorporating such formulation adjustments into clinical practice could be a simple yet effective strategy to improve the overall success of PN filler treatments.

## References

[1] He X, Gao X, Xie W. Research progress in skin aging and immunity. Int J Mol Sci. 2024;25:4101–14. 38612909 10.3390/ijms25074101 PMC11012511

[2] Choi EH. Aging of the skin barrier. Clin Dermatol. 2019;37:336–45. 31345321 10.1016/j.clindermatol.2019.04.009

[3] Galeano M, Bitto A, Altavilla D, Minutoli L, Polito F, Calo M. Polydeoxyribonucl-eotide stimulates angiogenesis and wound healing in the genetically diabetic mouse. Wound Repair Regen. 2008;16:208–17. 18318806 10.1111/j.1524-475X.2008.00361.x

[4] Lee DW, Hong HJ, Roh H, Lee WJ. The effect of polydeoxyribonucleotide on ischemic rat skin flap survival. Ann Plast Surg. 2015;75:84–90. 25954843 10.1097/SAP.0000000000000053

[5] Noh TK, Chung BY, Kim SY, Lee MH, Kim MJ, Youn CS. Novel anti-melanogene-sis properties of Polydeoxyribonucleotide, a popular wound healing booster. Int J Mol Sci. 2016;17:1448–58. 27598132 10.3390/ijms17091448 PMC5037727

[6] Lee YJ, Kim HT, Lee YJ, Paik SH, Moon YS, Lee WJ. Comparison of the effects of polynucleotide and hyaluronic acid fillers on periocular rejuvenation: a randomized, double-blind, split-face trial. J Dermatolog Treat. 2022;33:254–60. 32248707 10.1080/09546634.2020.1748857

[7] Kim JH, Kim ES, Kim SW, Hong SP, Kim J. Effects of polynucleotide dermal filler in the correction of crow’s Feet using an Antera three-dimensional camera. Aesthet Plast Surg. 2022;46:1902–9. 10.1007/s00266-022-02832-8 35357558

[8] Lee D, Kim MJ, Park HJ, Rah GC, Choi H, Anh ST. Current practices and perceived effectiveness of polynucleotides for treatment of facial erythema by cosmetic physicians. Skin Res Technol. 2023;29:e13466. 37753681 10.1111/srt.13466 PMC10485387

[9] Araco A, Araco F, Raichi M. An exploratory study of PN HPT for treating postsurgical atrophic and depressed scars. J Cosmet Dermatol. 2025;24:e16764. 39812340 10.1111/jocd.16764 PMC11734378

[10] Yi KH, Winayanuwattikun W, Kim SY, Wan J, Vachatimanont V, Putri AI. Skin boosters: definitions and varied classifications. Skin Res Technol. 2024;30:e13627. 38481069 10.1111/srt.13627 PMC10938033

[11] Hong JY, Lee YH, Kim H, Park KY. Therapeutic performance of needle injection versus needle-free jet injector system for polynucleotide filler in skin rejuvenation. J Cosmet Dermatol. 2025;24:e16595. 39370844 10.1111/jocd.16595 PMC11743049

[12] Smith L, Cockerham K. Hyaluronic acid dermal fillers: can adjunctive lidocaine improve patient satisfaction without decreasing efficacy or duration? Patient Prefer Adherence. 2011;5:133–9. 21448297 10.2147/PPA.S11251 PMC3063660

[13] Usach I, Martinez R, Festini T, Peris JE. Subcutaneous injection of drugs: literature review of factors influencing pain sensation at the injection site. Adv Ther. 2019;36:2986–96. 31587143 10.1007/s12325-019-01101-6 PMC6822791

[14] Best CA, Best AA, Best TJ, Hamilton DA. Buffered lidocaine and bupivacaine mixture – the ideal local anesthetic solution? Plast Surg. 2015;23:87–90. 10.4172/plastic-surgery.1000913 PMC4459414 26090348

[15] Jeong GJ, Ahn GR, Park SJ, Hong JY, Kim BJ. A randomized, patient/evaluator-blinded, split-face study to compare the efficacy and safety of polycaprolactone and polynucleotide fillers in the correction of crow’s feet. J Cosmet Dermatol. 2020;19:1593–9. 31680395 10.1111/jocd.13199

[16] Lowe PL, Lowe NJ. Botulinum toxin type B: pH change reduces injection pain, retains efficacy. Dermatol Surg. 2005;31:874–8. 10.1097/DSS.0000000000000178 25350125

[17] Brazeau GA, Cooper B, Svetic KA, Smith CL, Gupta P. Current perspectives on pain upon injection of drugs. J Pharm Sci. 1998;87:667–77. 9607942 10.1021/js970315l

[18] Berteau O, Filipe-santos O, Wang T, Rojas HE, Granger C, Schwarzenbach F. Medical devices: evidence and research. Med Devices Evid Res. 2015;8:473–84. 10.2147/MDER.S91019 PMC4646585 26635489

[19] Felton LA. Essentials of pharmaceutics. 1st ed. Boca Raton, FL: CRC Press, USA; 2013.

[20] Hijazi LO, Alghonaim Y, Alraee SA, Alqubaisy Y. Injection site pain, onset and duration of action of botulinum toxin reconstituted in normal saline with and without sodium bicarbonate: a prospective, single-center, randomized, double-blind interventional study. Plast Surg. 2021;1:1–6. 10.1177/22925503211011971 PMC9389062 35990391

[21] Oh YJ, Lee NY, Suh DH, Koh JS, Lee SJ, Shin MK. A split-face study using botulinum toxin type B to decrease facial erythema index. J Cosmet Laser Ther. 2011;13(5):243–8. 21848448 10.3109/14764172.2011.613479

[22] Kawane K, Motani K, Nagata S. DNA degradation and its defects. Cold Spring Harb Perspect Biol. 2014;6:a016394. 24890510 10.1101/cshperspect.a016394 PMC4031964

[23] Lauková L, Konečná B, Janovičová L, Vlková B, Celec P. Deoxyribonucleases and their applications in biomedicine. Biomol. 2020;10(7):1036. 10.3390/biom10071036 PMC7407206 32664541

